# Critically appraised topic (CAT) groups to improve the capability of healthcare staff to translate research findings into practice: A critical reflection

**DOI:** 10.1111/hir.12577

**Published:** 2025-06-17

**Authors:** Liz Lees‐Deutsch, Abby Kendrick

**Affiliations:** ^1^ Research and Development Centre for Care Excellence, University Hospitals Coventry and Warwickshire NHS Trust Coventry West Midlands UK; ^2^ Centre for Healthcare Communities Coventry University Coventry UK; ^3^ Library and Knowledge Services Clinical Evidence Based Information Service, University Hospitals Coventry and Warwickshire NHS Trust Coventry West Midlands UK

**Keywords:** collaboration, critical appraisal, database searching, education and training, evidence‐based practice (EBP), health professionals, informationist, librarians, translation of research findings

## Abstract

**Background:**

The development of a Centre for Care Excellence at a large Midlands National Health Service teaching hospital enabled the opportunity to introduce Critically Appraised Topic (CAT) groups through collaborative working with library specialists and health professionals.

**Objectives:**

To provide interactive training for health professionals to improve their critical appraisal skills and to translate research findings into practice.

**Methods:**

Clinical Evidence Based Information Services library experts and a clinical academic facilitator ran interactive CAT groups via webinars. Clinical staff were recruited via poster advertising with quick‐response (QR) code registration. Groups were facilitated for 8 months.

**Results:**

Between January 2019 and August 2023, six CAT groups were established. Four groups completed critical appraisal, progressing to translate the research findings to inform clinical practice. Progression paused in two groups, with outcomes reporting to follow.

**Discussion:**

CATs can galvanise health professionals' database searching, evidence retrieval, and critical appraisal; particularly those less familiar with these processes. Group members must commit to deliverables, especially with challenging workforce shortfalls where CAT groups could be designated as optional activities.

**Conclusions:**

Outcomes depend on the adequacy of critical appraisal skills and the involvement of skilled facilitators. Long‐term, a strategy to cultivate new facilitators through training may ensure scale‐up for new groups.

**Key Messages**

- *Users*: understanding ‘what works’ in CAT groups enables information specialists to provide targeted and informed guidance and support to clinicians seeking evidence‐based information.
- *Advocacy*: evidence on the impact of CAT groups can demonstrate how information specialists contribute to evidence‐based practice and patient care outcomes.
- *Professional development*: understanding barriers and facilitators to successful CAT groups enables information specialists to adapt their skills and knowledge to meet clinicians' evolving needs.
- *Collaboration*: theorising collaboration demonstrates how the pooling of expertise and resources can lead to more rigorous and comprehensive responses to clinical questions.

## BACKGROUND

One of the biggest challenges for health professionals is translating research findings into clinical practice. Developing the capability and capacity of health professionals to regularly engage with emerging evidence remains challenging (Jones & O'Connor, 2024). Passive dissemination is largely ineffective, and numerous conditions are necessary for the successful implementation of best evidence. Growing the capacity of healthcare organisations to deliver high quality evidence‐based care requires a continuous process of staff participation using proactive approaches (Jones & O'Connor, 2024; Kislov et al., 2014).

At University Hospitals Coventry and Warwickshire NHS Trust (UHCW), Critically Appraised Topic (CAT) groups are being introduced through a supportive facilitation approach, involving collaboration between a clinical academic (LLD) in the Centre for Care Excellence (CfCE) and Clinical Evidence Based Information Services (CEBIS) Specialist staff. The Centre for Evidence‐Based Management (CEBMa, 2017) provides a definition which states;

‘A Critically Appraised Topic group provides a quick and succinct assessment of what is known (and not known) in the scientific literature about an intervention or practical issue by using a systematic methodology to search and critically appraise primary studies’ [p. 3].

The CAT process was first developed at Keele University as a collaborative exercise between clinical teams, academics, and information‐based services to critically appraise evidence to determine ‘a clinical bottom line’ for practice (Stevenson et al., 2007, p. 704). Employing systematic review techniques, CATs adopt a streamlined and pragmatic approach by engaging the expertise of multiple healthcare professionals, generating practical responses to address the ‘so what?’ question for clinical applications (Finney et al., 2016). CATs work at expedited speed through collective engagement of health professionals in the critical appraisal process to discern a best evidence response to a clinical question (CEBMa, 2017; Keele University, 2023). They are regarded as being a good way to get a ‘quick impression’ of the evidence and although they are a group process, differ from Rapid Evidence Assessment in resource, skills and quality of evidence produced (CEBMa, 2017, p. 31). CAT group work proved very useful in involving nurses in reviewing evidence during the COVID‐19 pandemic regarding the best method giving important drugs to patients in need (Finney, 2022). Where speed of completion is of essence, for example, the release of staff from clinical duties is constrained due to workforce issues, namely recruitment and vacancies, CATs provide an option for healthcare staff to be involved in evidence generation, in preference to handing over a ‘literature search and review’ to information specialists.

There are a variety of different approaches for training health professionals (including didactic, interactive, and blended) and the demand for innovative evidence‐based training methods is increasing (CEBMa, 2017; Hecht et al., 2016). Typically, training is undertaken through passive involvement and is not instigated as a continuous practice (Kislov et al., 2014). Implicit in a CAT is the collaborative effort among clinical, academic and library staff, with expert facilitation and support throughout the process (Stevenson et al., 2021). Successful groups comprise ‘working’ health professional members who are prepared to commit to the deliverables, focused on the translation of the identified research findings into practice. Owing to their knowledge of the clinical domain, healthcare professional group members are well placed to discern the most relevant evidence, while academic expertise is essential to critically appraise evidence quality. Group membership is usually heterogenous and spans multiple disciplines, allowing decisions and outputs to be informed by diverse viewpoints on the topic. The process typically involves real‐time database searching.

To date, several CAT groups have been developed, each addressing clinical questions pertinent to a specific clinical context and topic/area. Prior to the development of CAT groups, the CEBIS team were actively engaged in establishing the evidence for clinicians, usually through literature searches, graded by evidence and through evidence in practice groups. While this work continues, the development of CAT groups aims to enable healthcare staff to proactively engage with evidence and co‐produce evidence, to be applied in practice. It is not the primary intention of CATs to find a gap in the literature; future research questions have been generated by the groups, addressing issues where evidence is sparse. CAT groups have also prompted changes to clinical practice and guidelines, challenged clinical assumptions, and averted the initiation of non‐evidence‐based service developments.

This article critically reflects on the experience of establishing CAT groups, commencing in 2019, and the current approach adopted in a large NHS trust (1000+ beds) in England since 2022. The issues encountered and key success factors for training health professionals in evidence assessment and implementation are discussed.

## OBJECTIVES

Our objectives were to promulgate the establishment of CAT groups across a large NHS Trust by providing training as a vehicle for healthcare professionals to conduct critical appraisal. We hoped members of new CAT groups formed would be equipped to translate their research findings into clinical practice. Essential to this was the provision of facilitation to support CAT groups through the process. This work fitted within the broader remit of the Centre for Care Excellence to translate research into practice to further enhance patient care and academic excellence, aligning with its themes of research, innovative practice, and professional education.

## METHODS

### Launch

In 2022, CAT groups were launched at UHCW through a training webinar delivered by CEBIS staff and a CfCE clinical academic, both with significant CAT methodology expertise and experience. Posters advertising the webinar were displayed in the hospital library, and digital information was also disseminated to all hospital staff via regular staff bulletins. Expressions of interest were then received via email from clinical practice leads and healthcare staff (physios and nurses); preprepared information was sent to these persons. Delegates then self‐enrolled for the training webinar using a quick‐response (QR) code. During the webinar, the trainers emphasised the participatory aspect expected from potential CAT groups members and typical membership, plus the anticipated timespan for completion of the work. Two options for participation were offered, namely a train the trainer model, where healthcare professionals acting as clinical champions could lead and grow the CAT groups in their own clinical areas, or CAT groups would be facilitated through ongoing supportive facilitation via CEBIS and academic support. Following the webinar, enquiries were received from clinical practice leaders keen to establish CAT groups in their respective areas.

**FIGURE 1:**
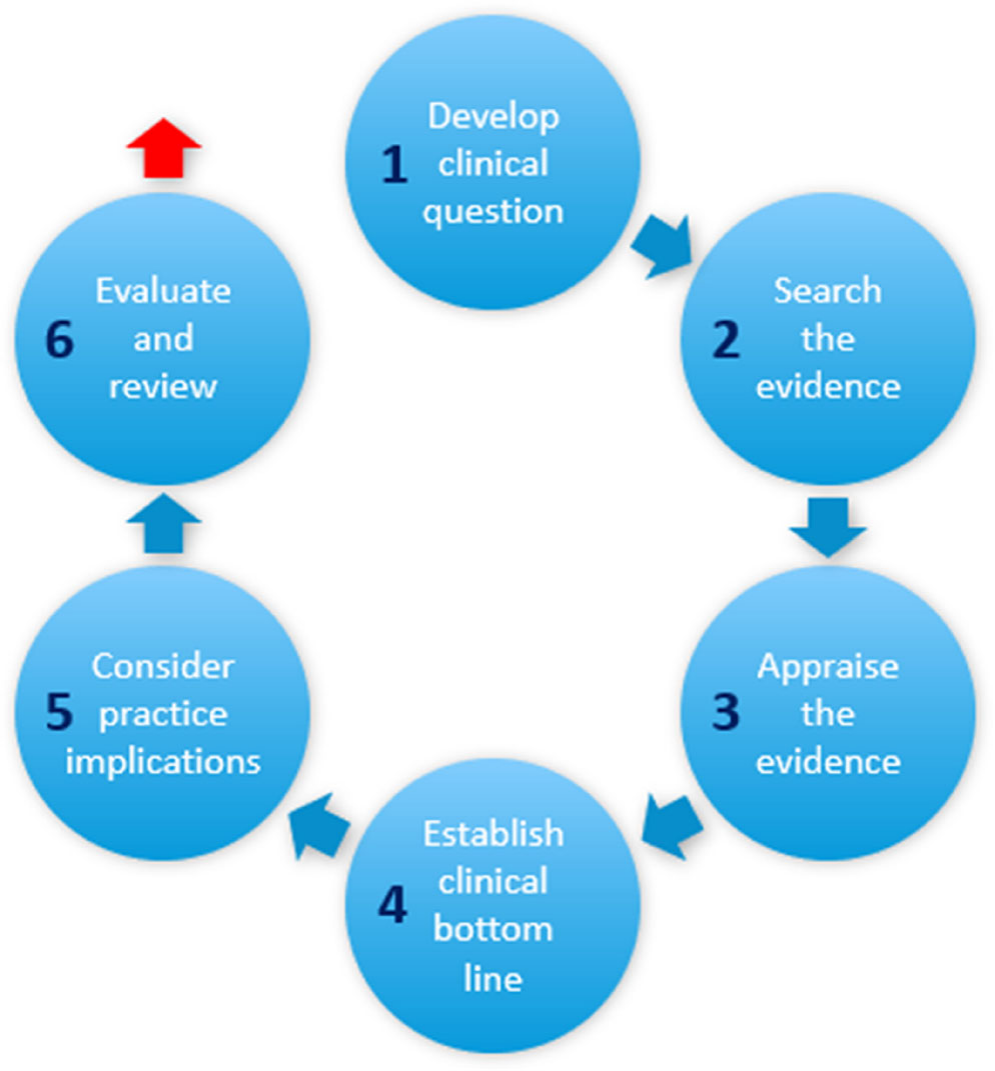
Stages of the critically appraised topic group process. [Colour figure can be viewed at wileyonlinelibrary.com]

The membership for each group was nominated by the clinical practice leads and guided throughout by the CAT facilitators in accordance with equality, diversity and inclusion principles. Of those staff nominated by clinical leads and self‐nominated, no one was excluded. Groups were comprised of emerging scholars—with motivations for further study; staff identified as benefitting from mentoring and development—to develop skills with using the evidence base and recent newly registered/recruited staff to the area. We believed a combination of staff would serve to motivate and encourage each other throughout the process. Three CAT groups were subsequently launched through clinical nomination and self‐nomination of staff. The launch of each CAT group commenced with an overview presentation of the CAT process, which set out key information about the methodology, stages of progression and processes involved (Figure 1).

### Delivering groups

The philosophy underpinning CAT groups aligns with the theory of constructivism, where learners construct or make their own knowledge and understanding through collaboration with others (Dewey, 1938; Fox, 2001). Another core principle of the approach relates to learning being an active process through social activity (group learning). Each CAT group is contextually associated with a clinical area, with the main goal being to translate key relevant evidence into practice. Since there are no prescriptive requirements for CAT group members' knowledge, assumptions are inevitably made regarding the adequacy of the individuals' understanding of the research processes involved. Engaging with the CAT process allows members to rekindle any prior knowledge in this regard and helps to foster greater understanding. The interactive element of CATs also encourages peer support and informal collaborations among group members, both within the CAT group and later when producing deliverables as part of a social partnership. Finally, the clinically driven review question, which addresses a specific practice problem, serves as both the context and motivating factor for completing the work.

A fundamental component of the CAT model is early involvement of a CEBIS expert, who collaborates closely with the clinical practice lead (s) to gain an appreciation of the clinical topic and project scope. In each of the groups, a CEBIS expert participated (either virtually through a digital link, e.g., Microsoft Teams©, or in person) to develop the search strategy and refine the search topic leading to the review question. Subsequent refinements to the search strategy were communicated to group members via email in between scheduled meetings. This approach demanded a significant time investment per meeting, each lasting up to 2 h, with an estimated total time commitment of 21 h throughout the entire CAT process, accounting for additional time spent by the CEBIS expert to refine the searches. Nevertheless, the timeliness of the work and sustained engagement is imperative to maintain momentum, and ultimately to ensure prompt availability of the review findings to practitioners.

We established that the process of screening and critically appraising articles can be completed in a day or staged over several meetings; this depends on the model preferred alongside the availability and preferences of group members and facilitators. The staged approach, which enables screening and critical appraisal to be completed more progressively, was employed across the groups and spanned an average timeframe of 8 months for completion of the work. Early decisions regarding members' preferred model helped to maintain the enthusiasm generated by discussions around pervasive clinical issues and ultimately expedited the generation of evidence‐based answers. This approach also allowed informal and ad‐hoc mentoring to evolve naturally during and between meetings, which created opportunities for members to build their confidence in applying research techniques through mutually reinforcing learning circles. All meetings were held in a location to suit the needs of clinical participants: for example, a co‐located education centre and meeting rooms within clinical departments.

## RESULTS

Based on our involvement with the facilitation of each group we can comment upon the characteristics of the membership. We did not, however, set out to collect data during our endeavours [in group facilitation], to this end we have reflected upon the motivations of each CAT group membership, as a homogenous unit. We acknowledge that individual members, may have had both personal (individual) and group aspirations.

### Characteristics of CAT groups

The CAT groups described here include three that were developed through prior work in another NHS Trust (2019–2021) and three from UHCW NHS Teaching Trust (2022–2024). Membership of groups was made up of healthcare staff nominations by clinical leads of services and self‐nominations by staff, resulting in the heterogenous characteristics described in Table 1.

**TABLE 1:** Topic, membership, and status of CAT groups.

Topic	Membership	Status according to stages (1–6; see Figure 1)	Year
Developing a high‐dependency unit on an acute medicine unit	Advanced clinical practitioners, trainee clinical practitioners, acute medicine consultant, critical care consultant	Completed up to considering practice implications (stage 5) [Outcome was that a high‐dependency unit was deemed to be financially unviable; no examples of staffing model]	2019
Reducing interruptions during medication rounds on the acute medicine unit	Pharmacist, matron, ward nurses, quality improvement lead	Completed searches and partial evidence appraisal (Stage 3) [Outcome was completed by group but not reported to CAT facilitator]	2019
Reasons for patient self‐ discharge on a newly developed ambulatory unit	Patient safety team, matron, consultant nurse	Completed up to considering practice implications (stage 5) [Outcome was updated information for patients]	2020
Developing dietary advice for gestational diabetes patients	Dieticians, nurses, consultant Psychologist	Completed up to establishing clinical bottom line (stage 4) [Outcome was information concluded]	2022
Mechanisms to reduce post‐operative delirium in cardiothoracic patients	Consultant, critical care nurses	Completed searches and partial evidence appraisal (Stage 3) [Outcome pending]	2022
Establishing the evidence to underpin delirium bundle components	Falls expert, lead nurses, physiotherapists	Completed up to searches and partial evidence appraisal (stages 2–3) [Outcome pending]	2023

### Motivations of CAT group members

The typical motivation of a CAT group is to ‘establish the bottom‐line evidence’ ready for translation into clinical practice which serves as a good starting point to draw the interest of health professionals (Stevenson et al., 2007, 2021). In two of the groups described here, staff joined as a step‐up from intra‐departmental journal clubs, seeing the CAT process as a new mechanism for achieving improved clinical outcomes in their departments. Other motivations described by group members included the need for continuing professional development, preparation for professional revalidation, and an interest in doing future research. These aspects were supported by the provision of attendance certificates, and encouragement of structured reflection on learning following the completion of CAT group activities.

Beyond personal motivations, several group members highlighted the potential benefits of CAT outputs, namely a summary report and presentation, in readiness for using as evidence to adapt clinical practice or develop new services. In cases where the clinical area was ‘research active’, CAT outputs were used as background information to support grant applications for related funding and future research proposals. In one instance, where the evidence generated by the CAT group clearly indicated a proposed clinical service development could be fraught with issues, the implementation was halted. This saved valuable time and NHS resources for clinical staff, who were able to revisit their options in the light of the new evidence from the topic group. Finally, we learnt that several healthcare staff wanted to understand the reasons for clinical variations in treatment with a view to standardising the care given. They felt that reading evidence by comparison to assessing evidence as a group process was more credible and gave them confidence to present the evidence. Regardless of staff members' reasons for engaging with CATs, the offer of wide‐ranging encouragement through facilitation, from the outset may help to retain momentum and interest in the outputs (Kislov et al., 2014).

This information was gathered through discussions with group members at the stage of final meetings. CAT group members were not followed up after the completion of a topic group, as resources beyond the establishment of groups and subsequent facilitation were not identified at the outset. Hence, CAT members were lost to follow up in understanding any other outcomes from their participation.

## DISCUSSION

The collaborative and social engagement of group members, together with the judicious involvement of a CEBIS expert, ensures the relevance of the clinical topic and availability of diverse expertise, both of which are needed to complete the work. Collaborating to co‐create the review strategies and outputs was anecdotally reported by group members as being preferable to working alone, both in terms of expediting and group learning from the process (Fox, 2001). The completion of work may also need to be galvanised by a facilitator. For example, while group members were encouraged to share any challenges they encountered and resolve issues through discussion, in the case of two groups, the facilitator was enrolled to independently screen and critically appraise the evidence in order to expedite the workload. While this was an amicable arrangement and welcomed by group members, it constrained the tacit learning within the group.

The need to provide ongoing teaching for health professionals involved in CAT groups was initially not recognised during the webinar launch. However, our experience of working with these groups has demonstrated that skilled leadership and sufficient time allocation for facilitation are imperative, particularly to support apprehensive group members (Stevenson et al., 2021). For example, regardless of members' enthusiasm, some nervousness around the CAT process was common, particularly in relation to critical appraisal. Each group was distinctive, and this was especially true with regard to prior experience of review skills. Some groups comprised members with no recent experience in using any of the key research skills, where confidence‐building exercises were required prior to active participation in group work. Others could initiate the development of a review question and undertake critical appraisal with minimal support, akin to phases 3–5 of knowledge‐based expertise on the Vitae Researcher Development Framework (2011).

Initially, many health professionals across the groups described feeling ‘bruised’ by their prior academic studies, which had caused a reluctance to get involved in research activities such as, evidence appraisal. While this apprehension influenced some members' level of engagement with the process, it diminished as they gained insight and clarity regarding their roles within the CAT group context. As facilitators, our natural response was to adopt a pragmatic approach, incorporating subtle strategies to revive and nurture members' research skills, and in some cases, to support the development of new ones. Without this approach, we have reflected that the work completed by busy clinical practitioners during the time between meetings would likely not have been possible. What is clear is that as new topics are explored, staff with varying degrees of skill and experience are likely to engage with the CAT process, and this will require a commitment from facilitators to continuously recognise and cultivate the necessary skills among group members.

In our experience, CAT groups take time to mature. Groups transition through stages such as forming, storming, norming and performing (Tuckman, 1965), and effective facilitation plays a crucial role in guiding this progression by providing clarity and ensuring smooth coordination of activities. This mediation helps members to build trust in one another and encourages self‐teaching within groups by way of virtuous learning circles. Moreover, facilitation that is based on a set of core values—consistent with the concepts of empowerment, commitment, collaboration, learning and partnership, or what Roger Schwarz describes as “facilitative leadership” (Schwarz, 2002, pp. 327–343)—creates the potential for group members to *practise* leadership, which in turn creates the conditions for groups to become self‐sustaining (Kislov et al., 2014). Members of a successful CAT group should, therefore, be ready to assume a leading role within a new group, thus freeing the facilitator to restart the process with a new topic. Hence, retaining some core members will not only build tacit knowledge but also help to make the model sustainable (Hecht et al., 2016). Nevertheless, the reality in acute healthcare settings, given the high staff turnover, it is unlikely that long lasting ‘communities of practice’ as seen in primary care, will develop (Finney, 2022).

Our experience reflects other findings in the literature, where the relational functions of initiating and facilitating group learning have been identified as key to developing research capacity and capability among health professionals (Flenady et al., 2022; Mickan et al., 2019, 2022; Ritchie et al., 2021). At the same time, there is clearly still some way to go. In a recent systematic review on the barriers, enablers, and strategies adopted by nurses and allied health professionals when embedding research into practice, Smith and Johnson (2023) identified three overarching and interconnected themes: leadership, capabilities, and organisation culture. Given that barriers, enablers, and strategies are ‘inherently intertwined’, the authors argue that successful strategies will require a whole organisational approach (p. 18). Access to information and academic support transpired in discussions with CAT members, this was expressed in general terms from ‘where to start’ to ‘how to write up’ work. Hence, an evidence strategy would need to include the necessary leadership to create opportunities for staff beyond CAT groups for mobilisation of evidence.

### Critical success factors

If CAT groups are to become embedded into the culture of an organisation, they should not be overly onerous for any members, and could be managed as task and finish groups. For example, each new topic presents the opportunity to reset and revisit group membership, with no obligation for members to continue onto a new topic. In one CAT group described here, members were keen to proceed to a new topic while they were ‘on a roll’, feeling comfortable with the experience and wishing to reapply their learning. On the other hand, should most members choose to opt out as topics and questions change, the withdrawal of tacit knowledge may affect any future cascading or scale‐up of the CAT model.

Importantly, our facilitation approach is not one‐size‐fits‐all, and facilitators must simultaneously attend to participants as individuals and members of a group. The model is therefore less predictable than more traditional didactic approaches, and there is no universal formula for success. As this approach is more likely to challenge habits and push participants and facilitators beyond their comfort zones, those involved must be willing to embrace change, self‐reflection, trust, and mutual respect. Such behavioural changes will encourage a transition from individual learning to a dynamic group activity where self‐confidence and skills contribute to organisational learning (Kislov et al., 2014). While engaging in CAT groups offers a practical opportunity for members to establish these skills, the effort required for success should not be underestimated, particularly within busy acute settings. Organisational support (typically time out) is therefore essential for establishing an environment where staff can develop these capabilities.

The CAT model does not end with critical appraisal, as depicted in Figure 1. A report needs to be compiled, framed around the original question to present the bottom‐line evidence, which then aids the dissemination and translation of research findings (Stevenson et al., 2007) (see Table 1). To progress beyond critically appraising individual articles associated with knowledge production (Greenhalgh et al., 2016) to the coherent application of bottom‐line evidence is a huge leap without sufficient skills in evidence interpretation and synthesis. Working through skill development requires that the skills required, which may be across several domains are identified (Vitae RDF, 2011). Indeed, synthesising evidence using various review methodologies is critical if this process is to be robust. While this process may not be the responsibility of a facilitator, or of library services, such as CEBIS, clinical leadership within each CAT group is essential to ensure ownership of the process throughout. This aspect required most support in each group, and perhaps remains the most challenging barrier to scaling up CAT groups via facilitation. Even though library experts such as CEBIS Specialists work with healthcare professionals to form the right question, compile the evidence search and facilitate critical appraisal, it is usually done on an individual basis. Information professionals need to utilise their knowledge mobilisation skills to facilitate this group learning.

## CONCLUSION

Our experience suggests that CAT group members expect to be taught on the various elements of undertaking a group evidence review. Through facilitation and expertise, this CAT group model ensures tangible outputs derived from the evidence, which are then translated into clinical practice through effective knowledge sharing. Nevertheless, the taught element requires further quantification and testing, which will require a change of approach to incorporate this. Empirical work is required to identify the baseline knowledge of group members and to test an adapted CAT model inclusive of teaching, based on our experience of facilitating groups through the Keele University model. We believe that this will gradually enable a transition from individual and group activities towards ongoing sustainable organisational learning.

## FUTURE RESEARCH

We propose to continue our work based on the reflections of our experience, using a before and after study approach to test the feasibility of an adapted CAT which is promoted as a staff development opportunity.

## FUNDING INFORMATION

No funding has been received for this work; it was carried out as part of roles at the affiliations stated.

## CONFLICT OF INTEREST STATEMENT

I am the corresponding author and I advise that the authors have no personal interests or conflict of interest to declare relating to the publication of the submitted manuscript.

## Data Availability

There is no data—this is a reflective piece of work.

## References

[hir12577-bib-0001] Center for Evidence‐Based Management . (2017). CEBMa Guideline for Critically Appraised Topics in Management and Organisations. https://cebma.org/assets/Uploads/CEBMa-CAT-Guidelines.pdf (Accessed: 24 September 2024).

[hir12577-bib-0002] Dewey, J. (1938). Experience and education. Macmillan Company.

[hir12577-bib-0003] Finney, A. G. (2022). Promoting true evidence‐based practice using critically appraised topics (CATs). Blog: Evidence Based Nursing (Accessed: 24 September 2024).https://blogs.bmj.com/ebn/2022/10/09/promoting-true-evidence-based-practice-using-critically-appraised-topics-cats/

[hir12577-bib-0004] Finney, A. G. , Johnson, K. , Edwards, J. , Duffy, H. , & Dziedzic, K. (2016). Critically appraised topics (CATs): A method of integrating best evidence into general practice nursing. Practice Nurse, 46, 32–34.

[hir12577-bib-0005] Flenady, T. , Dwyer, T. , Kahl, J. , Sobolewska, A. , Reid‐Searl, K. , & Signal, T. (2022). Research capacity‐building for clinicians: Understanding how the research facilitator role fosters clinicians' engagement in the research process. Health Research Policy and Systems, 20(1), 45. 10.1186/s12961-022-00849-8 35477479 PMC9044663

[hir12577-bib-0006] Fox, R. (2001). Constructivism examined. Oxford Review of Education, 27(1), 23–35.

[hir12577-bib-0007] Greenhalgh, T. , Jackson, C. , Shaw, S. , & Janamian, T. (2016). Achieving research impact through Co‐creation in community‐based health services: Literature review and case study. The Milbank Quarterly, 94(2), 392–429. 10.1111/1468-0009.12197 27265562 PMC4911728

[hir12577-bib-0008] Hecht, L. , Buhse, S. , & Meyer, G. (2016). Effectiveness of training in evidence‐based medicine skills for healthcare professionals: A systematic review. BMC Medical Education, 16(1), 103. 10.1186/s12909-016-0616-2 27044264 PMC4820973

[hir12577-bib-0009] Jones, B. , & O'Connor, C. (2024). Promoting evidence‐based practice and nursing excellence: How involvement in a Magnet4Europe® research study led to development of critically appraised topics sessions for health care staff. Health Information and Libraries Journal, 41(1), 109–112. 10.1111/hir.12504 37606075

[hir12577-bib-0010] Keele University . (2023). Evidence into Practice Groups: Allied Health Professions. https://www.keele.ac.uk/iau/evidenceintopracticegroups/alliedhealthprofessions/#!

[hir12577-bib-0011] Kislov, R. , Waterman, H. , Harvey, G. , & Boaden, R. (2014). Rethinking capacity building for knowledge mobilisation: Developing multilevel capabilities in healthcare organisations. Implementation Science, 9(1), 166. 10.1186/s13012-014-0166-0 25398428 PMC4234886

[hir12577-bib-0012] Mickan, S. , Hilder, J. , Wenke, R. , & Thomas, R. (2019). The impact of a small‐group educational intervention for allied health professionals to enhance evidence‐based practice: Mixed methods evaluation. BMC Medical Education, 19(1), 131. 10.1186/s12909-019-1567-1 31060553 PMC6503357

[hir12577-bib-0013] Mickan, S. , Wenke, R. , Weir, K. , Bialocerkowski, A. , & Noble, C. (2022). Using knowledge brokering activities to promote allied health clinicians' engagement in research: A qualitative exploration. BMJ Open, 12(4), e060456. 10.1136/bmjopen-2021-060456 PMC9058767 35487731

[hir12577-bib-0014] Ritchie, M. J. , Parker, L. E. , & Kirchner, J. E. (2021). From novice to expert: Methods for transferring implementation facilitation skills to improve healthcare delivery. Implementation Science Communications, 2(1), 39. 10.1186/s43058-021-00138-5 33832549 PMC8033694

[hir12577-bib-0015] Schwarz, R. M. (2002). The skilled facilitator: A comprehensive resource for consultants, facilitators, managers, trainers, and coaches (2nd ed.). John Wiley & Sons.

[hir12577-bib-0016] Smith, S. , & Johnson, G. (2023). A systematic review of the barriers, enablers and strategies to embedding translational research within the public hospital system focusing on nursing and allied health professions. PLoS One, 18(2), e0281819. 10.1371/journal.pone.0281819 36795679 PMC9934318

[hir12577-bib-0017] Stevenson, K. , Bird, L. , Sarigiovannis, P. , Dziedzic, K. , Foster, N. E. , & Graham, C. (2007). A new multidisciplinary approach to integrating best evidence into musculoskeletal practice. Journal of Evaluation in Clinical Practice, 13(5), 703–708. 10.1111/j.1365-2753.2006.00715.x 17824861

[hir12577-bib-0018] Stevenson, K. , Sarigiovannis, P. , Finney, A. G. , Cottrell, E. , Lewis, R. , Edwards, J. J. , Hadley‐Barrows, T. , Thomson, K. , Reay, H. , & Dziedzic, K. S. (2021). Development, spread and impact of primary care and musculoskeletal communities of practice to assist rapid translation of evidence into practice. Musculoskeletal Care, 19(4), 564–569. 10.1002/msc.1552 33755287

[hir12577-bib-0019] Tuckman, B. W. (1965). Developmental sequence in small groups. Psychological Bulletin, 63(6), 384–399. 10.1037/h0022100 14314073

[hir12577-bib-0020] Vitae Researcher Development Framework . (2011). Careers Research Advisory Centre. www.vitae.ac.uk/vitae‐publications/rdf‐related/researcher‐development‐framework‐rdf‐vitae.pdf/view (Accessed: 20 February 2024).

